# Experimental Infection of Horses with Influenza D Virus

**DOI:** 10.3390/v14040661

**Published:** 2022-03-23

**Authors:** Chithra C. Sreenivasan, Tirth Uprety, Stephanie E. Reedy, Gun Temeeyasen, Ben M. Hause, Dan Wang, Feng Li, Thomas M. Chambers

**Affiliations:** 1 Maxwell H. Gluck Equine Research Center, University of Kentucky, Lexington, KY 40546, USA; chithra.sreenivasan@uky.edu (C.C.S.); tirth.uprety@uky.edu (T.U.); sereed0@uky.edu (S.E.R.); dan.wang@uky.edu (D.W.); feng.li@uky.edu (F.L.); 2 Department of Veterinary and Biomedical Sciences, South Dakota State University, Brookings, SD 57007, USA; gun.temeeyasen@sdstate.edu (G.T.); benjamin.hause@sdstate.edu (B.M.H.)

**Keywords:** influenza D, horses, pathogenic, type D, seroconversion, bovine IDV

## Abstract

Antibodies to influenza D virus (IDV) have been detected in horses, but no evidence of disease in the field has been reported. To determine whether IDV is infectious, immunogenic, and pathogenic in horses, four 2-year-old horses seronegative for both influenza A (H3N8) and D viruses were intranasally inoculated with 6.25 × 10^7^ TCID_50_/animal of D/bovine/California/0363/2019 (D/CA2019) virus, using a portable equine nebulizer system. Horses were observed daily for clinical signs including rectal temperature, nasal discharge, coughing, lung sounds, tachycardia, and tachypnea. No horses exhibited clinical signs of disease. Nasopharyngeal swabs collected from 1–8 days post-infection demonstrated virus shedding by qRT-PCR. The horses showed evidence of seroconversion as early as 13 days post-infection (dpi) and the geometric mean of the antibody titers (GMT) of all four horses ranged from 16.82–160 as demonstrated by the microneutralization assay. Further, deep RNA sequencing of the virus isolated in embryonated chicken eggs revealed no adaptive mutations indicating that IDV can replicate in horses, suggesting the possibility of interspecies transmission of IDV with bovine reservoir into equids in nature.

## 1. Introduction

Equine influenza is prevalent in most of the world causing a significant economic burden to the equine industry. Among the four influenza types, equines are affected by type A, which is a highly contagious respiratory disease spreading through aerosol, contact, and fomite, and the main subtypes involved are H3 and H7 [1]. Of these, H7 is reportedly extinct, and the H3 subtype diverged into several lineages, sub-lineages, and clades, associated with equine infections globally. Both H3 and H7 subtypes have been evolved from influenza viruses of avian origin. Equine influenza A virus (EIV-A) causes nasal discharge, fever, cough, lethargy, and loss of appetite with occasional lower respiratory complications due to secondary bacterial pneumonia.

Apart from EIV-A, influenza D virus with bovines as reservoir host were found to have circulated in the midwestern equine populations [2]. Apart from horses, the influenza D virus exhibits a broad host range which includes pigs, sheep, goats, camels, and humans [3,4,5,6,7,8]. A seroepidemiological study involving 464 samples collected from the equine farms/ranches from Iowa, Minnesota, North Dakota, Nebraska, South Dakota, and Wyoming in 2015 revealed 11–12% seroprevalence against the two predominant IDV lineages (D/OK and D/660) in horses. However, another epidemiological study for IDV in equines from the UK using sera and respiratory samples from upper respiratory tract (URT) infections with unknown causes indicated seropositivity in 1/330 samples by hemagglutination inhibition (HI) and 6/430 samples by pseudo-type virus neutralization test (PVNT) [9].

Although several hosts have been identified for the IDV, little is known about the susceptibility of horses against IDV. The serological and genomic evidence of IDV in occupational workers working with swine and human environments have been documented [6,8,10,11,12,13,14]. Horses have been so closely associated with humans for centuries, and it would be worthwhile to test the susceptibility of IDV in horses. The 9-O-acetylated sialic acids were considered influenza D receptors and the distribution of the acetylated sialic acids is one of the host determinants for the tropism. Previous studies have shown that horse erythrocytes demonstrated higher Neu5Gc expression with diverse 9-O-acetyl modifications [15]. Further, in vitro virus binding assays on equine respiratory tissues also revealed positive binding in the submucosal glands, nasal, and pharyngeal epithelia, which suggests that IDV could replicate in horses [16,17]. This study was aimed to investigate the susceptibility of the equids to IDV by studying the clinical manifestations, virus shedding, and also the ability to undergo seroconversion after experimental exposure to a bovine originated IDV isolate (D/CA2019).

## 2. Materials and Methods

### 2.1. Cells and Viruses

Madin-Darby canine kidney (MDCK) cells were maintained in Dulbecco’s minimum essential medium supplemented with 10% fetal bovine serum (FBS) (PAA Laboratories Inc., Dartmouth, MA, USA) and penicillin-streptomycin (Life Technologies, Carlsbad, CA, USA) (100 U/mL) were used in this study for propagating cell and viral cultures. The IDV strain used for the study was influenza D/bovine/California/0363/2019 virus. The virus was propagated on MDCK cells. Following infection, fresh DMEM with 1 μg/mL tolyl-sulfonyl phenylalanyl chloromethyl ketone (TPCK)-treated trypsin (Sigma, Saint Louis, MO, USA) was added and incubated at 33 °C in 5% CO_2_ for 5 days. The supernatant was spun at 500× *g* for 10 min at 4 °C to remove the cellular debris. The virus titers were determined using MDCK cells according to the method of Reed and Muench [18]. Virus loads in nasopharyngeal swabs were also titrated using MDCK cells. DMEM supplemented with penicillin-streptomycin (Life Technologies, Carlsbad, CA, USA) (200 U/mL) and TPCK-treated trypsin (Sigma, Saint Louis, MO, USA) (1.0 μg/mL) was used as the virus growth and maintenance medium.

### 2.2. Animals and Experimental Design

Four horses of 2 years age, seronegative for IAV and IDV were used for the IDV challenge study. The duration of the experiment was 60 days, which included a 1-week acclimatization period. All four animals were intranasally inoculated with 6.25 × 10^7^ TCID_50_/2.5 mL of influenza D/bovine/California/0363/2019 (D/CA2019) virus, using a Flexineb (Flexineb, Union City, TN, USA) portable equine nebulizer system, a method that has been regularly employed to produce infection in experimental studies of equine influenza A virus [19]. To test the growth kinetics of the virus, nasopharyngeal swabs were collected from the animals from 0–8, and 13-days post-infection (dpi). The animals were regularly monitored for clinical signs and changes in body temperature, nasal discharge, coughing, palpation of lymph nodes, lung and gut sounds by auscultation, tachycardia, and tachypnea. To test the seroconversion, sera were collected from all the animals on 13, 21, 42, and 60 dpi. The animal experiments were conducted under biosafety level 2 conditions, according to the Institutional Animal Care and Use Committee approved protocol no: 2007-0153 of the University of Kentucky.

### 2.3. Estimation of Viral Shedding by 50% Tissue Culture Infectivity Dose (TCID_50_) Infectivity Assay and qRT-PCR

Virus loads in nasopharyngeal swab samples were titrated using MDCK cells. Serial ten-fold dilutions of the nasal samples prepared in DMEM supplemented with 1% penicillin-streptomycin and 1.0 μg/mL TPCK-treated trypsin (Sigma, Saint Louis, MO, USA) were transferred on pre-seeded MDCK cell culture plates. The plates were incubated at 33 °C for 5 days. The virus titers were determined according to the method of Reed and Muench [18].

Viral RNAs were extracted from the samples using the PureLink™ RNA Mini Kit (Invitrogen, Waltham, MA, USA) according to the manufacturer’s instructions. RT-PCR was performed using the High-Capacity RNA-to-cDNA™ Kit (ThermoFisher Scientific, Waltham, MA, USA) as per the instructions. PB1 gene was used for primer designing and a TaqMan probe was also used for detecting D/CA2019 in nasopharyngeal swabs. The primers used were Forward: 5′-AAAATTCATCGCTGTTTGCA-3′, reverse: 5′-TAACTCCAAGGCTATGTTTGA-3′, and probe 6FAM-CACCCATAGGATTTTTCCAAAGA-MGBBHQ. The qRT-PCR assay was performed using ViiA 7 Real-Time PCR instrument (Applied Biosystems technologies, Waltham, MA, USA) and the thermal cycling conditions used were 50 °C for 2 min; 95 °C for 20 s; 40 cycles of 95 °C for 1 s at, 60 °C for 20 s. The cut-off Ct value of the RT-qPCR assay was 39.

### 2.4. Virus Culture in Embryonated Chicken Eggs, Genome Sequencing and Analysis

The nasopharyngeal swabs positive for D/CA2019 virus at 3 dpi were inoculated into embryonated chicken eggs as described previously [20]. The egg culture positive for D/CA2019 virus was sequenced using an Illumina MiSeq system as described previously [21]. Contigs were assembled de novo using CLC Genomics and analyzed by BLASTX using the BLAST2Go plugin and the non-redundant protein sequence database [22]. The nucleotide sequences of the whole genome of D/CA2019 recovered from the egg culture were analyzed against the homologous sequences of D/bovine/California/0363/2019 in the NCBI database using the standard nucleotide BLAST [23].

### 2.5. Determination of Antibody Titers by Microneutralization Assay

The microneutralization (MN) assays were performed as described in the WHO standard manual [24] and a previous study [2]. MDCK cells were used for the MN assay. For the MN assay, serial 2-fold dilutions of serum samples were tested in duplicate. The MN titers were expressed as the reciprocal of the highest dilution of serum showing complete inhibition of virus. The presence and absence of the virus were determined by hemagglutination assay using 1% turkey red blood cells (Lampire Biological Laboratories, Pipersville, PA, USA). All samples were assayed in four separate experiments and the geometric mean antibody titers (GMT) were calculated.

## 3. Results

### 3.1. Clinical Outcomes and Viral Shedding

**Figure 1:**
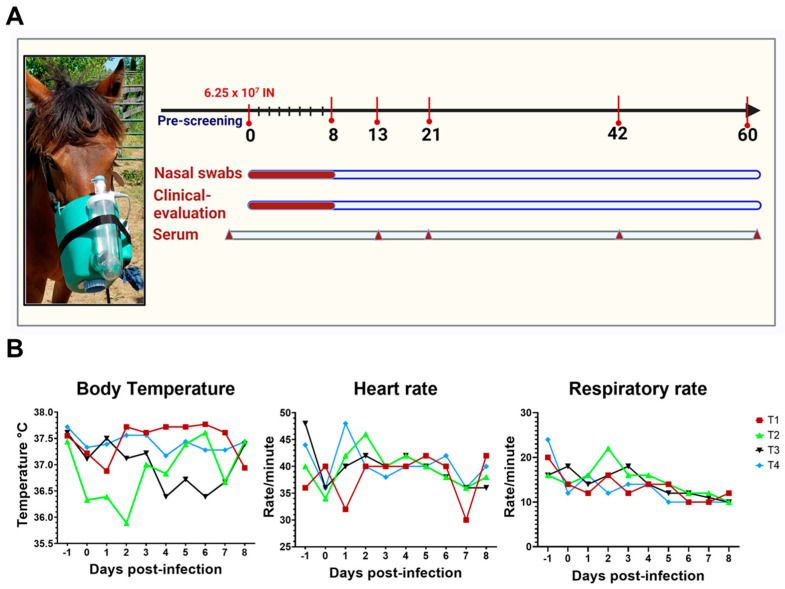
Experimental design and clinical outcomes of the D/CA2019 virus in horses. (**A**) Experimental plan—four 2-year-old horses (T1, T2, T3, and T4), seronegative for both influenza-D virus and also type A (H3N8) equine influenza, were intranasally inoculated with 6.25 × 10^7^ TCID_50_/animal of influenza D/bovine/California/0363/2019 (D/CA2019) virus, using Flexineb, a portable equine nebulizer system. Clinical, virological, and serological parameters of infection were assessed following intranasal inoculation of D/CA2019. The clinical evaluation included the presence of influenza-like illness, nasal discharge, changes in body temperature, heart rate, respiratory rate, changes in the peripheral lymph nodes of the four horses, monitored for 8 days (shown in red highlights). To study the viral shedding, nasopharyngeal swabs were collected from 0 to 8 dpi and sera were collected at 13, 21, 42, and 60 dpi to check seroconversion. (**B**) Changes in the temperature, heart rate, and respiratory rate of the four horses. Each shape represents an individual animal. (T1: red square, T2: green triangle, T3: black inverted triangle, T4: blue diamond). The graphical illustration was created with BioRender.com, accessed on 28 January 2022.

The horses after intranasal inoculation did not show overt clinical signs. The animals were regularly monitored for changes in body temperature, the color of the gums, changes in the peripheral lymph nodes (submandibular, parotid) by palpation, lung sounds by auscultation, gut sounds, tachycardia, and tachypnea. The horses, upon intranasal challenge, did not develop any respiratory signs characteristic of influenza. There were no significant changes in body temperature, heart, and respiratory rates compared to the basal temperature, heart, and respiratory rates before infection (Figure 1B). The animals were also monitored for changes in the peripheral lymph node, any nasal discharge, and changes in the fecal characteristics, and mild changes were noticed in the clinical parameters in one or two animals. Slight serous discharge was noticed once in three of the four horses (T2, T3, and T4) on 5–7 dpi (Figure S1, Supplementary Data). Only one of four horses (T4) showed slight enlargement of the parotid glands from 5–8 dpi. The lung auscultation, gut sounds, and fecal consistencies of all the horses were within normal limits post-infection (Figure S1).

To check the viral shedding of D/CA2019 post-infection, nasopharyngeal swabs were collected from 1–8 dpi. The nasopharyngeal swabs failed to give appreciable titers by 50% tissue culture infective dose assay (TCID_50_) on MDCK cells which have a detection limit (100 TCID_50_/_mL_). Further, the viral shedding in nasopharyngeal swabs was estimated by performing a standard RT-qPCR assay targeting the PB1 gene of D/CA2019 (Table 1). D/CA2019 virus was detected in all the animals from 1–8 dpi as demonstrated by the Ct values ranging between 31–39 (the cut-off Ct: 39). Appropriate positive and negative controls were included in the PCR assay. To further confirm the viral shedding, representative nasopharyngeal samples from 3 dpi from all four animals were inoculated into the allantoic cavity of the embryonated chicken eggs for virus isolation. Out of the four animals, the T4 sample yielded virus with 32 HA (5 log_2_) units. To detect whether D/CA2019 evolved to acquire any adaptive mutations, the full-length genome of this nasal swab-derived virus in egg culture was determined by deep RNA sequencing. Full genome sequence analysis demonstrated that D/CA2019 recovered from the eggs did not undergo any adaptive mutations. This indicates that IDV can successfully adapt and replicate in the equine respiratory tract as indicated by the positive Ct values of the nasopharyngeal swabs from RT-qPCR, virus isolation in chicken eggs, and whole-genome sequencing results.

### 3.2. Seroconversion

**Figure 2:**
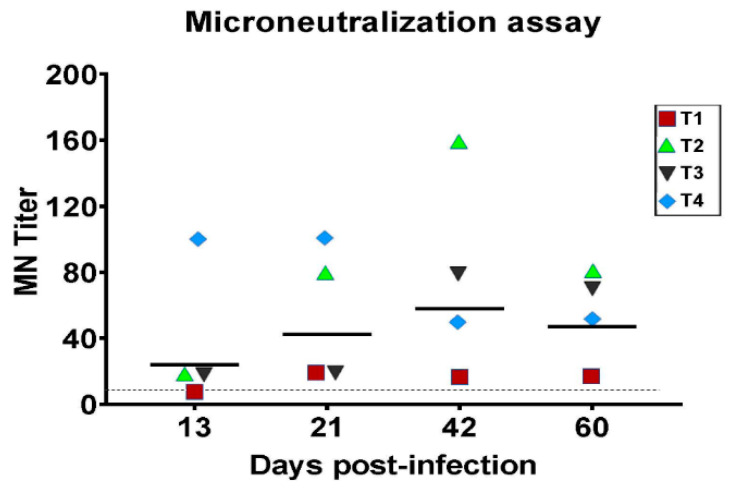
Seroconversion of D/CA2019 in horses. The D/CA2019 specific viral titers were determined by the MN assay using MDCK cells. Each shape represents an individual animal. The log-transformed values of individual antibody titers (GMT) are shown (T1: red square, T2: green triangle, T3: black inverted triangle, T4: blue diamond) with a horizontal geometric-mean bar shown at 13, 21, 42, and 60 dpi. A dotted line indicating the limit of detection is also shown.

To check seroconversion, pre and post-infection sera were tested for virus-specific antibodies by microneutralization assay. All four animals were seronegative to influenza A and D antibodies before the infection. Sera were collected from all the animals on 13, 21, 42, and 60 dpi. On 13 dpi, three out of four animals demonstrated antibody titers with GMT ranging between 20 and 95.14. Interestingly, all the animals seroconverted with titers ranging from 20–160 on 21, 42, and 60 dpi. The individual MN titers for all four animals are shown in Figure 2. The antibody titers increased at 21 and 42 dpi with a GMT of 41.77 and 56.57, respectively, and then decreased to 45.55 at 60 dpi.

## 4. Discussion

Influenza D viruses with bovines as the primary reservoir have spilled over to other species such as swine, small ruminants, camelids, and horses across the world. Besides bovines, influenza D viruses were also isolated from swine and caprine [5]. Apart from these agricultural animals, influenza D was found to have a high seroprevalence in the camelids, particularly in dromedary camels in Africa [4]. Serological evidence of IDV was also found in the mid-western equine populations of the USA against the two IDV lineages, D660 and DOK [2]. Recent reports on the serological evidence of IDV in humans particularly in swine veterinarians and other occupational workers [6,7,10,11] are intriguing. Horses have been closely associated with humans for a long time, but little is known about the natural susceptibility of horses to IDV. Previous in vitro studies have shown that horses possess 9-O-acetylated sialic acids in the upper respiratory tissues [25]. Further, horse RBCs can agglutinate two IDV lineages (D660 and DOK), indicating that IDV receptors are present in the horse RBCs [16]. The histochemical staining of the equine nasal and pharyngeal epithelium indicated that both D660 and DOK can bind to the submucosal glands of the nasal epithelium and pharyngeal epithelia [16]. However, another virus binding study performed on the respiratory tissues from bovine, sheep, goats, horses, swine showed attachment only to the submucosal glands of the horses and not to the nasal turbinate, pharynx, trachea, and lung [17]. In the light of these facts, we conducted a proof-of-concept experiment, to test the susceptibility of horses to IDV.

It is worthwhile to note that the virus we used for the challenge is a recently isolated IDV strain, D/bovine/California/353/2019, belonging to the new phylogenetic lineage [26]. We used this IDV strain for the experimental challenge firstly, because D/CA2019 virus-specific rabbit polyclonal sera exhibited broad neutralizing activities against D660 and DOK lineage viruses in vitro [26]. Since the co-circulation of D660 and DOK lineage viruses in mid-west equines was already known, it would be interesting to check the susceptibility of the equines against this recent isolate. Second, most of the IDV isolates of US origin belong to the D660 and DOK lineages [27]. The antigenic and phylogenetic characterization studies have shown that D/CA2019 belongs to a new phylogenetic lineage and possesses broad antigenicity [26]. Third, the percentage identity of the amino acid sequence of hemagglutinin esterase fusion (HEF) protein of D/CA2019 to D660 and DOK is 95.63 and 95.48 respectively [26]. Considering the percent of sequence identity between the HEF protein of these three lineages, we speculated that D/CA2019 could replicate in horses.

The experimental inoculation of 10^7^ TCID_50_/10 mL of D/bovine/C00046N/Mississippi/2014 intranasally in the principal host species, involving four months old calves also demonstrated viral shedding in nasal swabs and upper respiratory tissues by RT-qPCR, contact transmission, and seroconversion. Infected calves, however, only showed mild clinical signs. This study used younger animals (4-months old), despite the similar infection dose used in our study [28]. This bovine infection study also did not find changes in the body temperature, heart rate, and respiratory rate, which is consistent with our findings. However, another experimental study in calves using D/bovine/France/5920/2014 demonstrated moderate clinical signs and pathology in the upper and lower respiratory tract and demonstrated transmission by contact and aerosol [29]. For our study, we did not collect respiratory tissues, so the pathology and tissue tropism is not known. The findings of our study have shown that the horses, after infection with 6.25 × 10^7^ TCID_50_/animal of D/CA2019, did not show any overt clinical signs. However, the exposed horses demonstrated nasal shedding and also developed IDV-specific antibodies. We used a flexineb portable nebulizer to intranasally inoculate the animals, which has been used regularly to produce infection in experimental studies of the equine influenza A (H3N8) virus [19]. After infection, changes in temperature, body weight, heart rate, respiratory rate, lymph node changes, lung auscultation, gut changes, etc. were closely monitored for 8 days post-infection and compared to pre-infection readings. None of the horses showed changes in their vital signs. Slight serous nasal discharge was noticed only once in three of the four horses from 5–7 dpi, while the lower respiratory tract (LRT) signs indicated by the abnormal lung auscultatory sounds were not found in any of the four horses during the clinical evaluation period. The animal T4 exhibited slight enlargement of the parotid gland from 5–8 dpi, and the virus isolation from nasal swab at 3 dpi from this animal yielded a high titer. Further, this animal seroconverted with an individual higher MN titer with a GMT of 95.14 as early as 13 dpi, while other horses demonstrated titers ≤20. Compared to horses, the experimental infection of D/bovine/C00046N/Mississippi/2014 in calves caused seroconversion with a higher hemagglutination inhibition titer of 640 at 21 dpi [28]. The virus antibody titer above 40 is considered the gold standard for influenza. In our experiment, three out of four animals demonstrated MN titers ranging from 80–160 by 42 dpi. Our previous seroprevalence study in mid-western equine populations in the USA has shown 12% seroprevalence against D660 and DOK lineage viruses and the titers ranged from 10–160. Interestingly, we found comparable titers with a peak titer of 160 in our study similar to the peak titers observed for the field samples [2]. The evidence of nasal shedding combined with successful virus isolation in embryonated chicken eggs followed by the recovery of the whole genome of D/CA2019 from the nasal swab derived egg culture indicates that the virus productively replicated in the horse respiratory tract and did not acquire any adaptive mutations to become replication-competent under experimental settings. The findings of our in vivo study also corroborate the positive binding of D660 and DOK hemagglutinin esterase protein to equine submucosal glands and URT tissues in vitro [16]. There are not many equine IDV surveillance studies, which limits our knowledge of the global scenario regarding the natural exposure of the equines to IDV. A recent screening study of 232 respiratory samples from equids with suspected URT disease in the UK, was negative, indicating that IDV is not associated with respiratory disease in horses [9]. Serological screening of thoroughbreds in training and breeding stock also revealed a low level of seroprevalence, 1/330 by HI and 6/430 by PVNT [9]. Several studies have been performed both in vivo and in vitro using D660 and DOK lineage viruses. D/CA2019, being a contemporary strain, has not been well-characterized, and hence its receptor binding preferences and species specificities are not known and need further investigation.

In summary, the findings of our study indicate that bovine origin IDV did not cause any respiratory disease in equids; however, the virus can replicate in the respiratory tract and horses undergo successful seroconversion after experimental exposure, which suggests that the interspecies transmission of IDV to equids can occur in nature. In the USA, we have IDV strains largely isolated from bovines compared to swine, but swine were the most affected species in Eurasia [27]. Recent evidence suggests that IDV is also circulating in feral swine populations in the United States [30]. The species specificity is an important factor governing the cross-species transmission and it was already reported that interspecies transmission between calves and pigs was dependent on the species-specific IDV strain [31]. In the light of the findings of our study, the susceptibility of horses to IDV needs further investigation in different lineages, as slight phenotypic characteristics can change the species preferences and cross-species transmission, which in turn can affect the viral ecology. Hence, more studies are needed to study the species specificity, receptor distribution, and natural susceptibility of IDV in horses.

## Figures and Tables

**Table 1:** Detection of viral load in nasal swabs from DCA2019 infected animals by RT-PCR.

Days Post Infection	Mean Ct Values			
	T1	T2	T3	T4
0	nd	nd	nd	nd
1	37.35	34.42	nd	38.61
2	35.65	nd	nd	35.34
3	32.67	36.65	38.99	34.68
4	32.51	36.19	36.94	33.89
5	34.11	34.88	33.71	32.32
6	31.43	36.92	33.86	33.41
7	33.02	37.68	35.26	35.02
8	31.53	36.61	35.18	38.70

nd: not detected.

## Data Availability

The data presented in this study will be available on request from the corresponding author.

## Supplementary Materials

The following supporting information can be downloaded at: https://www.mdpi.com/article/10.3390/v14040661/s1, Figure S1: Illustration of clinical changes in horses after experimental infection of IDV.
