# Supplementary material for: Experimental Infection of Horses with Influenza D Virus
Source: Viruses. 2022 Mar 23;14(4):661. doi: 10.3390/v14040661 (PMC9029652; doi:10.3390/v14040661)
Supplement: Supplementary file 1 [file viruses-14-00661-s001.zip › viruses-1601496-supplementary.pdf]

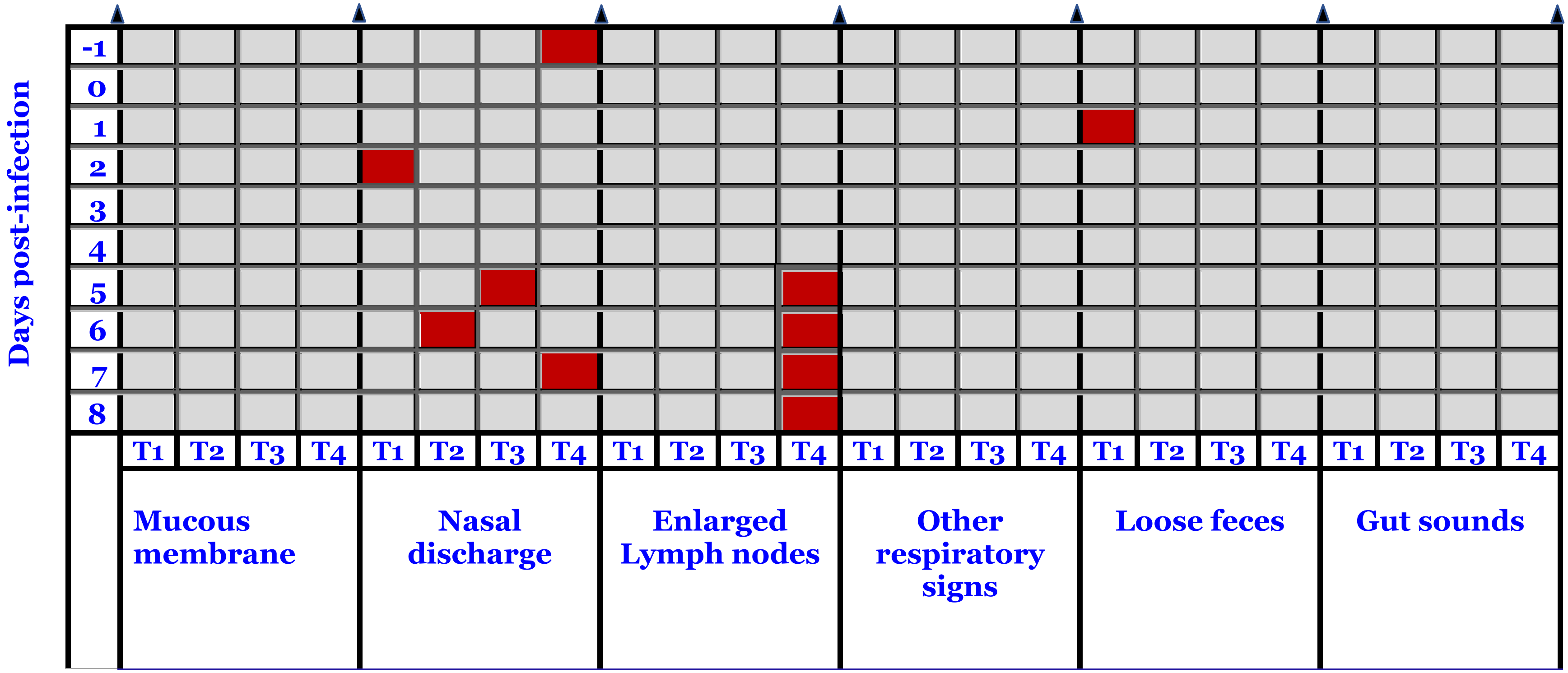

Figure S1: Illustration of the clinical changes observed in the horses after experimental inoculation of IDV. The days post-infection are illustrated on the Y-axis and clinical signs on the X-axis. Clinical changes monitored included mucous membrane changes, nasal discharge, lymph node enlargement, other respiratory signs such as coughing, wheezing, pulmonary auscultatory changes, GI changes such as peristalsis, and fecal consistency. Red: present; grey: absent (within normal limits).
